# Diagnostic validity of the anxiety and depression questions from the Well-Being Process Questionnaire

**Published:** 2018-06-29

**Authors:** Gary Williams, Andrew P Smith

**Affiliations:** ^1^Centre for Occupational Health Psychology, School of Psychology, Cardiff University, UK

**Keywords:** diagnostic validity, anxiety, depression, well-being, WPQ, hospital anxiety and depression scale

## Abstract

**Background:**

Previous research shows that the Well-being Process Questionnaire (WPQ) has good content validity, construct validity, discriminant validity and reliability.

**Aims:**

The present research examined the diagnostic validity of the anxiety and depression questions from the WPQ by comparing them with the Hospital Anxiety and Depression scale (HADS) from which they were derived.

**Method:**

One hundred and twenty university staff members aged 20-64 participated in the study which involved an anonymous online survey. The data were used to assess the ability of single item measures, rated on a 10 point scale, to correctly identify a respondent that meets a diagnostic criteria, in this case clinical levels of depression or anxiety.

**Results:**

This analysis involved comparison with an established measure (HADS clinical cut-off) in terms of the proportion of those with the condition correctly identified as such (sensitivity) by the single items and the proportion without the condition correctly identified as such (specificity) by the single items. The results showed that a cut-off point at a score of 5 provided the best results for sensitivity and specificity in the depression and anxiety items. Sensitivity at this point was 71.4% and 86.3% for depression and anxiety respectively, while specificity was 85.4% for depression and 72.6% for anxiety.

**Conclusion:**

These findings confirm that the single item anxiety and depression questions from the WPQ can be used as an initial screening tool to identify clinical cases of anxiety and depression.

**Relevance for patients:**

This will provide a rapid method of assessment that will benefit patients and lead to more effective prevention and management.

## 1 Introduction

The Well-Being Process Questionnaire (WPQ) is a mea-suring instrument that uses short versions of established ques-tionnaires to investigate factors which change well-being and well-being outcomes. Choice of variables has been linked to the Demands-Resources-Individual effects model [1] which has been validated using the original scales in studies with nurses and teachers [2,3]. Similar results have been obtained using the WPQ [4–6]. The aim of the present article is to focus on the anxiety and depression questions from the WPQ and examine their diagnostic validity. Diagnostic validity is one aspect of the psychometrics of the WPQ and it is important to initially briefly review other aspects of validity and reliability of the WPQ (see [7] for a more detailed account)

The validity of a measure is assessed in relation to its in-tended purpose [8,9]. Content and construct validity, which are concerned with how well the measure represents the variable for which it is designed, are of primary importance for outcome measures because the purpose of these measures is to accurately represent the variable in question [9]. Predictive validity is more important for predictor variables [8,9], as their purpose is to pre-dict scores on a particular outcome. The content validity of mea-sures should be ensured in the construction of the measure itself [9]. In previous research this has been achieved by sampling

items from a pool of already available items [10]. In the present research a similar approach was followed by sampling ques-tions from the multi-item measures into the examples provided alongside the single-item measures of the WPQ. Measures of abstract variables require construct validity, which is concerned with how well the results represent the behaviour of a person with the construct in question [8], therefore providing a proxy measure of a variable that cannot be measured directly. One ap-proach to this type of validation in a newly-designed measure is to compare it to an established measure of the same variable, referred to as concurrent validity when the measures are com-pleted at the same time [8]. The single-item measures from the WPQ were compared to established multi-item measures already in use, in order to establish their construct or concurrent valid-ity. From a statistical standpoint, correlations of.80 are used to consider two sets of scores as unidimensional [11]. However, correlations between two measures designed to assess the same construct are frequently lower due to attenuation [9]. For exam-ple, Costa and McCrae [12] found correlations of.65 for two ex-traversion measures and.68 for two neuroticism measures (Six-teen personality factor questionnaire and Eysenck personality in-ventory). This research suggests that moderate correlations are often found between accepted multi-item measures of the same construct and these standards can also be applied to single-item measures. The correlations between the anxiety and depression single items from the WPQ and the anxiety and depression scales from the HADS were both 0.65, suggesting good validity [13]. In addition, the single anxiety and depression items were shown to be sensitive outcomes of established predictor variables (work characteristics and personality, [4].

Reliability is possibly the most commonly criticized, yet in-frequently assessed, aspect of single-item measures [14]. This is most likely due to an assumed inability to estimate the internal consistency of a single-item, although multiple approaches do exist. One approach is based on the correlations between scores taken at 3 points in time [15] (Equation 9), however the most direct approach is using the Wanous [16,17] method, using the correction for attenuation formula. Using this formula, Wanous et al [17] estimated the minimum reliability of a single-item job satisfaction question as.70 based on a meta-analysis of 17 stud-ies. The estimated reliability (using the Wanous method) of the single item anxiety score was 0.66, and the reliability of the sin-gle item depression score was 0.61.

The aim of this article is to examine the diagnostic validity of the single item anxiety and depression variables in the WPQ. Di-agnostic validity refers to the ability of the measure to correctly identify a respondent that meets a diagnostic criterion, for exam-ple clinical levels of depression or anxiety. This method involves comparison with an established measure in terms of the propor-tion of those with the condition correctly identified as such (sen-

sitivity) and the proportion without the condition correctly iden-tified as such (specificity). This is most common in depression and anxiety research where specific criteria exist for diagnosis of clinical depression or anxiety. This is relevant to our aim of developing short, practical measures and the newly designed measures should identify those with high or low mental health accurately so that, when the measures are applied in practice, in-tervention is targeted at the right people. In the present study, the single-item depression and anxiety measures were compared against the HADS measures, which contain cut-off scores for cases of depression or anxiety and a ‘normal range’.

## 2. Methods

This research was approved by the Ethics committee, School of Psychology, Cardiff University, and carried out with the in-formed consent of the participants. Participants were recruited from the staff of Cardiff University using an advertisement on the university noticeboard. The study involved an online survey presented using SurveyTracker that they could complete in their own time. Participants were instructed that they could skip any questions that they were not comfortable answering, and all data were provided anonymously. Informed consent was achieved within the questionnaire where participants without agreeing could not continue beyond the consent page. Following the con-sent page, participants were presented with an instructions sheet and a debrief sheet.

### 2.1. Participation

One hundred and twenty university staff members aged 20-64 participated in the study. Participants from all areas of the university were able to participate, including finance, teaching, accommodation, and security, although the role of specific re-spondents was not recorded. The majority were aged 30-39 (32%), married or living with a partner (63%) and were edu-cated to degree or higher degree level (73%). Working patterns were most commonly full-time (81%) fixed hours (79%). This sample was considered representative of a typical UK university and the number in the sample allowed identification of a medium effect size.

### 2.2. Materials

The survey consisted of single items derived from estab-lished psychosocial scales. The complete questionnaire is de-scribed in detail in [6]. The key questions in the present analyses are the anxiety and depression variables which are shown below:

Anxiety: On a scale of one to ten, how anxious would you say you are in general? (e.g. feeling tense or ’wound up’, unable to relax, feelings of worry or panic)?

1= Not at all 10 = Extremely

Depression: On a scale of one to ten, how depressed would you say you are in general? (e.g. feeling ’down’, no longer look-ing forward to things or enjoying things that you used to)?

1= Not at all 10 = Extremely

The scores on these variables were compared with the anx-iety and depression scores from the Hospital Anxiety and De-pression Scale [18].

### 2.3. Analyses strategy

The diagnostic validity of the outcome measures was exam-ined by creating groups based on the distribution of scores on outcome measures using the single-and multi-item scales. Sen-sitivity and specificity were examined for depression and anxiety where the multi-item scale includes known cut-offs for cases of clinical depression or anxiety. Low and high scores based on the possible range on the single items for depression and anxiety were compared with established cut-offs for ‘no depression/anx-iety’ and ‘mild’ to ‘severe’ depression/anxiety.

## 3. Results

Table 1 shows the demographic charateristics of the sample.

Correlations between the short and long versions of the ques-tions were similar for the different demographic groups (e.g. married: depression *r* = 0.61, anxiety *r* = 0.49; single: de-pression *r* = 0.66, anxiety *r* = 0.49. Educated to degree level: depression *r* = 0.66, anxiety *r* = 0.51. Not educated to degree level: depression *r* = 0.51, anxiety *r* = 0.54).

**Table 1:** Demographic characteristics of the sample

Age	Mean 36.9 years s.d. 10.6 range 21-64 years
Gender	Male: 27.5%
Material status	Single: 31.7%
	Living with partners: 25.8%
	Married: 36.7%
	Separated/Divorced: 5.8%
Education:	To degree level: 73.3%
Ethnicity	White/British: 98.3%

Table 2 shows the sensitivity and specificity for the WPQ single item depression and anxiety values plotted against nor-mal and moderate/severe categories from the HADS. A cut-off point at a score of 5 provided the best results for sensitivity and specificity in the depression and anxiety items. Sensitivity at this point was 71.4% and 86.3% for depression and anxiety respec-tively, while specificity was 85.4% for depression and 72.6% for anxiety.

**Table 2:** Sensitivity and specificity for single-item measures compared to multi-item measures of depression and anxiety for each cut-off score on the single-item measure (bold=median score).

Depression			Anxiety		

Cut-off point	Normal range (1-7)	Mild-Severe (8+)	Cut-off point	Normal range (1-7)	Mild-Severe (8+)
9	-	-	9	98.4	0
8	100	4.8	8	98.4	11.8
7	95.1	19	7	98.4	35.3
6	91.5	33.3	6	85.5	64.7
5	85.4	71.4	5	72.6	86.3
4	78	6.2	4	62.9	90.2
3	65.9	90.5	3	48.4	98
2	43.9	90.5	2	32.3	100
1	8.5	100	1	8.1	100

## 4. Discussion

The present results show that single items measuring anx-iety and depression have good diagnostic validity. This, com-bined with good content validity, predictive validity and relia-bility, suggests that the WPQ can be used in applied situations such as an initial screening tool for mental health problems. This initial screening could even be done remotely (e.g. over the tele-phone or by e-mail) to reduce logistic problems associated with primary care appointments. Similarly, it has other practical fea-tures, such as being low cost, that make it a useful alternative to established methods. However, it should be emphasised that this initial screen would then lead to a more detailed consideration of the causes and prevention/management of the mental health problem. The WPQ, based on the adapted DRIVE model, can now be used in longitudinal studies and to evaluate interventions. These interventions could involve changes in job characteristics, development of coping skills or therapeutic approaches dealing with established problems. The presence of an underlying model and short measuring instrument will enable more effective pre-vention and management of negative influences and outcomes, and also allow promotion of positive well-being.

The present study has it’s limitations, most of which reflect the sample used. Future studies should investigate a sample which is more representative of the general population and also a sample of clinical patients. It is important to know whether sim-ilar results are obtained with the single items with groups who have less awareness of the concepts of anxiety and depression.

## 5. Summary

In summary, the present study has shown that the WPQ sin-gle items measuring anxiety and depression have good diagnos-tic validity. The use of these items enables one to assess com-mon mental health problems very quickly and the WPQ is an ideal tool to use in both audits of psychosocial factors and the assessment of interventions.

## Conflict of interest disclosure

The authors have nothing to disclose.
